# Bone scan findings of Paget’s disease of bone in patients with *VCP* Multisystem Proteinopathy 1

**DOI:** 10.1038/s41598-024-54526-7

**Published:** 2024-03-11

**Authors:** Rod Carlo Agram Columbres, Sarosh Din, Liliane Gibbs, Virginia Kimonis

**Affiliations:** 1 Division of Genetics and Genomic Medicine, Department of Pediatrics, University of California, Irvine, CA USA; 2 College of Osteopathic Medicine, William Carey University, Hattiesburg, MS USA; 3 Department of Radiology, University of California, Irvine, CA USA; 4 Department of Pathology, University of California, Irvine, CA USA

**Keywords:** Medical genetics, Neurological disorders, Neuromuscular disease

## Abstract

Multisystem Proteinopathy 1 (MSP1) disease is a rare genetic disorder caused by mutations in the Valosin-Containing Protein (*VCP*) gene with clinical features of inclusion body myopathy (IBM), frontotemporal dementia (FTD), and Paget’s disease of bone (PDB). We performed bone scan imaging in twelve patients (6 females, 6 males) with confirmed *VCP* gene mutation six (50%) of which has myopathy alone, four (33%) with both PDB and myopathy, and two (15%) were presymptomatic carriers. We aim to characterize the PDB in diagnosed individuals, and potentially identify PDB in the myopathy and presymptomatic groups. Interestingly, two patients with previously undiagnosed PDB had positive diagnostic findings on the bone scan and subsequent radiograph imaging. Among the individuals with PDB, increased radiotracer uptake of the affected bones were of typical distribution as seen in conventional PDB and those reported in other MSP1 cohorts which are the thoracic spine and ribs (75%), pelvis (75%), shoulder (75%) and calvarium (15%). Overall, we show that technetium-99m bone scans done at regular intervals are a sensitive screening tool in patients with MSP1 associated *VCP* variants at risk for PDB. However, diagnostic confirmation should be coupled with clinical history, biochemical analysis, and skeletal radiographs to facilitate early treatment and prevention complications, acknowledging its limited specificity.

## Introduction

*VCP* Multisystem Proteinopathy 1 (MSP1), also known as IBMPFD or *VCP* disease (OMIM #167,320), is a rare autosomal dominant genetic disorder that affects the muscle, bone, and the central nervous system. MSP1 is caused by missense mutations in the *VCP* gene on chromosome 9p13.3-12. More than 65 variants have been linked to this disease, with the R155H, R155C, and R191Q variants being the most frequent^1–3^. Clinical manifestations include progressive adult-onset proximal muscle weakness, vacuolar and inclusion body muscle pathology in 50%, *VCP* PDB reported in 40%, and impairments in cognitive, behavioral, and social graces associated with frontotemporal dementia in approximately 30% of MSP1 patients^1,2,4,5^.

PDB is the second most common bone remodeling disease after osteoporosis in the general population^6^. It has a general prevalence of 2.1% in people older than 40 years old, with a slightly higher occurrence in males, but present in 40% of individuals with *VCP* disease^1^. PDB is caused by abnormal bone homeostasis where disorganization of bone by overactive osteoclasts and osteoblasts leads to clinical features of bone pain, bone deformity, deafness, fractures, and rarely osteosarcoma. The detailed mechanism of PDB is currently inconclusive, however, a combination of genetic, viral, and environmental factors has been suspected. One of most important predisposing genetic factor is variants in the *SQSTM1* gene that causes osteoclast activation via RANK signaling in 5–20% of PDB patients^7,8^. Other causative genes of PDB include *VCP, PFN1, OPTN, CSF1, TNFRSF11A,* and *TM7SF4*^9–12^. Elevation of bone turnover biochemical markers such as serum alkaline phosphatase (ALP), are associated^13^, however, normal ALP levels are observed in early or localized PDB disease.

In MSP1, PDB is reported in approximately 40% of patients^14^. PDB is often asymptomatic and is found incidentally in imaging and lab studies, however bone pain can be observed in 30% of MSP1 patients^5^. PDB can manifest as the first symptom in 4.9% of patients^15^. Co-presentation of myopathy and PDB as the initial manifestation occurs in 16% of MSP1 patients^1^. There are no studies comparing the biochemical or clinical differences between the PDB in MSP1 and conventional PDB. The mechanism of PDB pathogenesis however is similar since previous studies have shown that pathogenic variants in *VCP* can upregulate the NF-kB signaling pathway leading to upregulated osteoclastogenesis and bone resorption^7^ and can therefore contribute to PDB^16–18^. Similarly, report of *VCP*’s role in osteoblast activity involves complex regulation of bone morphogenetic protein (BMP) receptors via the *VCP* mediated ubiquitin/protein degradation system which may play a part in PDB pathogenesis^19,20^. Despite the lack of treatment options for the neuromuscular and neurological manifestations of MSP1, treatment of PDB with bisphosphonates is very effective in alleviating bone pain, and preventing deformity and other comorbidities.

Generally, plain radiographs are used to diagnose PDB due to their ability to show cortical thickening, bone expansion, and bone deformity^21,22^. While plain radiographs have high specificity, their sensitivity for the disease is relatively low. Alternatively, skeletal scintigraphy or bone scans prove to be more sensitive diagnostic tests compared to radiographs, especially in assessing regions of asymptomatic disease, albeit with lower specificity^23^. Since technetium-99m tracers (Tc-99m) are absorbed intensely in areas with increased osteoblastic activity, it is used to identify bones at risk for local complications^23^. Early stages of PDB present as osteolytic lesions or advancing lytic wedges in long bones on plain radiographic imaging, while sclerotic changes associated with thickened trabeculae and cortices, bone expansion, and deformity are seen later. Currently, most bone scan data in MSP1 patients is scattered in different case reports. Thus far, no formal bone scan analysis on the disease extent has been performed. Here, we present bone scan findings from twelve patients with MSP1 from one clinical center and systematically reviewed the anatomically affected areas. We also discuss guidelines for diagnostic testing and treatment of PDB.

## Patients and methods

We recruited twelve patients (6 females and 6 males) from seven families with MSP1 and confirmed *VCP* gene mutation at the UC Irvine Medical Center (Table 1). Our patient cohort included affected individuals with *VCP* associated IBM only, both IBM and PDB, and carriers. Prior diagnosis of PDB was made by their physicians based on elevated serum ALP, with or without bone pain, and imaging findings. The carriers were diagnosed by presymptomatic testing for the familial variants. This study was approved by UC Irvine Institutional Review Board (IRB 2007-5832). All patients signed an informed consent. All methods were performed in accordance with the relevant guidelines and regulations.

**Table 1:** Clinical characteristics and comorbidities in all MSP1 patients included in the study.

ID	Age at visit	Sex	Protein variant	DNA variant	Clinical features at visit	Myopathy Dx age (yrs.)	PDB Dx age (yrs.)	ALP levels (44–147 IU/L)	CK levels (20–200 IU/L)	No. of bones with increased uptake	Comorbidities		
											Cardiomyopathy	ALS	Bone Pain
1	60	F	R155H	464G > A	IBM, PDB	33	37	72	155	8	−	−	+
2	43	M	R155H	454G > A	IBM, PDB	42	38	84	271	14	−	+	−
3	54	M	R155P	464G > C	IBM, PDB	48	37	143	191	8	−	−	−
4	49	M	I254F	760A > T	IBM, PDB	49	45	234	2378	5	−	−	−
5	57	M	R155C	463C > T	IBM	45	−	70	99	2	−	−	+
6	28	F	R155C	463C > T	IBM	25	28*	59	50	1	−	−	−
7	62	M	R155H	464G > A	IBM	33	−	77	61	4	−	−	−
8	59	F	R155H	464G > A	IBM	51	−	131	177	3	−	−	+
9	53	F	R155P	464G > C	IBM	50	−	108	94	4	−	−	+
10	38	F	R155H	464G > A	IBM	35	38*	42	87	3	−	−	−
11	36	F	R155C	463C > T	Presymptomatic	37^#^	−	62	90	5	+	−	−
12	35	M	R155C	463C > T	Presymptomatic	37^#^	−	64	104	3	+	−	−
Mean (± SD)	47.8 ± 11.0	6F/6 M	−	−	−	40.4 ± 8.0	37.2 ± 4.95	95.5 ± 50.7	313.1 ± 625.5	−	17%	8%	33%

Dx, diagnosis; IBM, inclusion body myopathy; PDB, Paget’s disease of the bone; PDB, Paget’s disease of the bone; “ + ”, presence of comorbidity.

[*]: PDB diagnosis at the time of bone scan visit.

[#]: Diagnosed at later age (after bone scan visit).

[−] data not available or not observed.

All laboratory findings from the patient’s blood and imaging presented here were performed at UC Irvine Medical Center, Orange, CA. Bone scintigraphy used to survey the whole skeleton and assess the extent of disease uses a radiotracer like technetium-99m diphosphonate (Tc-99m) to identify areas of increased bone activity. All participants received an intravenous injection of technetium-99m and were imaged after 3 hours for whole-body imaging in the anterior and posterior projections. Radiograph imaging was performed on the same day of the pertinent area if an abnormality was identified on the bone scan. All bone scans and radiographs were read and interpreted by a blinded board-certified radiologist, and a second review included the senior author. Assessment of PDB was correlated with clinical history, lab testing and radiographic findings. All statistical analysis was conducted using Microsoft Excel version 16.71 and Prism version 9.5.1, and *p*-values ≤ 0.05 was considered statistically significant.

## Results

Clinical characteristics are summarized in Tables 1 and 2. Four (33.3%, ID 1–4) had both myopathy and PDB at the time of visit with a mean age of PDB onset at 39.3 years [range, 38–45 years], and a mean age of myopathy onset at 43.0 years [range, 33–49 years]. Six (50%, ID 5–10) had myopathy only at the time of visit with a mean age of onset at 39.8 years [range, 25–51 years], in addition to two pre-symptomatic carriers (16.7%, ID 11–12). Two patients with myopathy only were diagnosed with PDB at the time of visit (ID 6 and 10) with bone scans and subsequent correlation with radiographs and lab findings. Bone scan findings in different anatomical locations for all individuals diagnosed with PDB are summarized in Table 3. Degenerative joint disease (DJD) was noted on bone scan in seven individuals including the two pre-symptomatic individuals. Only one patient known to have PDB had bone pain attributable to the PDB. No controls were included in this study because of the risks of exposure to radiation, as requested by the IRB. The two initially pre-symptomatic individuals later developed myopathy at age 37 respectively (ID 11 and 12), and also had cardiomyopathy associated with a pathogenic c.177_187del variant in the *MYBPC3* gene^24^.

**Table 2:** Summary of clinical data in MSP1, IBM only and PDB patients.

Characteristics	IBM (n = 6)	IBM and PDB (n = 4)	MSP1* (n = 10)	Pre-symptomatic (n = 2)	*P* value
Sex (F,%)	4 (67%)	1 (25%)	5 (50%)	1 (50%)	0.80^a^
Age at visit (yrs.)	49.5 ± 13.5	51.5 ± 6.3	50.3 ± 10.4	35.5 ± 0.5	0.98^b^
Age of myopathy dx (yrs.) Mean ± SD	39.8 ± 10.4	43.0 ± 6.2	41.1 ± 8.5	−	0.42^b^
Age of PDB dx (yrs.) Mean ± SD	−	39.3 ± 3.3	−	−	−
ALP levels (IU/L) Mean ± SD	81.2 ± 32.8	133.25 ± 64.1	102 ± 53.2	63.0 ± 1.0	0.16^b^
CPK levels (IU/L) Mean ± SD	94.7 ± 44.7	748.75 ± 941.6	356.3 ± 677.0	97.0 ± 7.0	0.25^b^

F, Female; MSP1, Multisystem proteinopathy-1; IBM, Inclusion body myopathy; PDB, Paget’s disease of the bone.

[*] analysis excludes presymptomatic patients.

[−] data not available or not observed.

^a^Chi-square test.

^b^Unpaired t test.

**Table 3:** Bone scan data in all MSP1 patients per major anatomical locations.

All bone scan uptake										Increased uptake due to DJD									Comments
ID	Skull	Lumbar	Thoracic/Ribs	Shoulder	Humerus	Femur/Tibia/Fibula	Pelvis	Clavicle	Foot	Skull	Lumbar	Thoracic/Ribs	Shoulder	Humerus	Femur/Tibia/Fibula	Pelvis	Clavicle	Foot	
1	** + **	** + **	** + **	** + **	−	−	−	** + **	−	−	** + **	** + **	** + **	−	−	−	** + **	−	Skull uptake consistent with PDB; other anatomical uptakes is consistent with DJD; Previously treated but still had active disease. Plain radiographs confirm bone scan findings in the skull and spine T10 (Fig. 1)
2	−	−	** + **	** + **	−	−	** + **	** + **	** + **	−	−	−	** + **	−	−	−	** + **	** + **	Pelvic and spine uptake consistent with PDB; Shoulder, clavicle, and foot uptake due to DJD. Plain radiographs confirm bone scan findings in pelvis and T12 (picture framing and enlargement of vertebral body)
3	−	−	−	** + **	−	−	** + **	−	** + **	−	−	−	** + **	−	−	−	−	** + **	Pelvic uptake consistent with PDB, DJD noted at intertrochanteric region, right first toe, left midfoot and shoulders
4	** + **	** + **	** + **	−	−	** + **	** + **	−	−	−	−	−	−	−	−	−	−	−	All uptakes consistent with PDB
5	−	−	** + **	−	−	−	−	−	−	−	−	−	−	−	−	−	−	−	Rib uptake due to uncertain etiology not typical of PDB
6	** + **	−	−	−	−	−	−	−	−	−	−	−	−	−	−	−	−	−	L superior parietal calvarium involvement due to Paget’s; PDB diagnosis while at visit (Fig. 4a)
7	−	−	−	−	−	−	−	−	−	−	−	−	** + **	−	−	−	−	−	Multiple joint uptakes due to DJD
8	−	−	** + **	−	−	−	−	−	−	−	−	** + **	−	−	−	−	−	−	Rib uptake due to DJD; later diagnosed with PDB
9	−	−	−	−	−	−	** + **	−	−	−	−	−	−	−	−	** + **	−	−	Uptake in this patient is due to DJD
10	−	−	−	−	−	−	** + **	−	−	−	−	−	−	−	−	−	−	−	Increased activity on the right ischium and acetabulum due to PDB diagnosed while at visit (Fig. 4b)
11	−	−	−	** + **	−	−	−	** + **	−	−	−	−	** + **	−	−	−	** + **	−	Clavicle and shoulder uptake due to DJD
12	−	−	** + **	−	−	−	−	−	−	−	−	−	−	−	−	−	−	−	Rib uptake with unknown etiology
% MSP1	25%	17%	50%	33%	0%	8%	42%	25%	17%	0%	8%	17%	42%	0%	0%	8%	25%	17%	−

DJD, Degenerative joint disease; MSP1, Multisystem proteinopathy-1; [+], observed findings; [−], no observed findings.

### Spine and ribs

**Figure 1:**
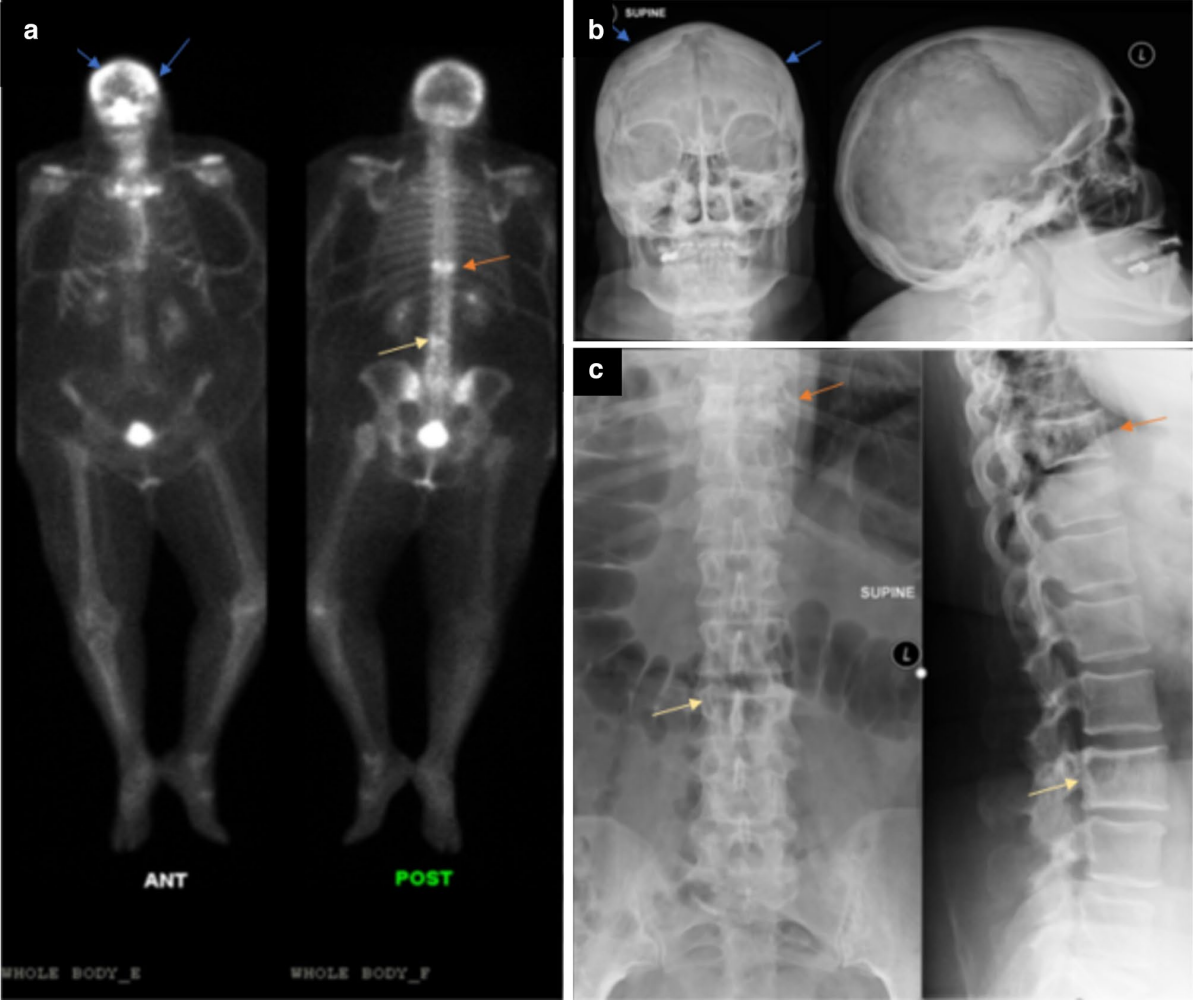
Whole body 99mTc-MDP bone Scan and Radiographic findings in patient 1 (ID 1). A 60-years-old female with back pain and leg pain, limb girdle distribution of myopathy and Paget disease revealed (**a**) increased uptake in the skull, thoracic and lumbar vertebrae on whole-body bone scan, increased uptake in the knees due to associated arthritis, (**b**) radiograph images reveal corresponding bone lesions of the skull bilaterally (blue arrows), (**c**) thoracic T10 vertebrae (orange arrow) and lumbar L3 vertebrae (yellow arrow).

**Figure 2:**
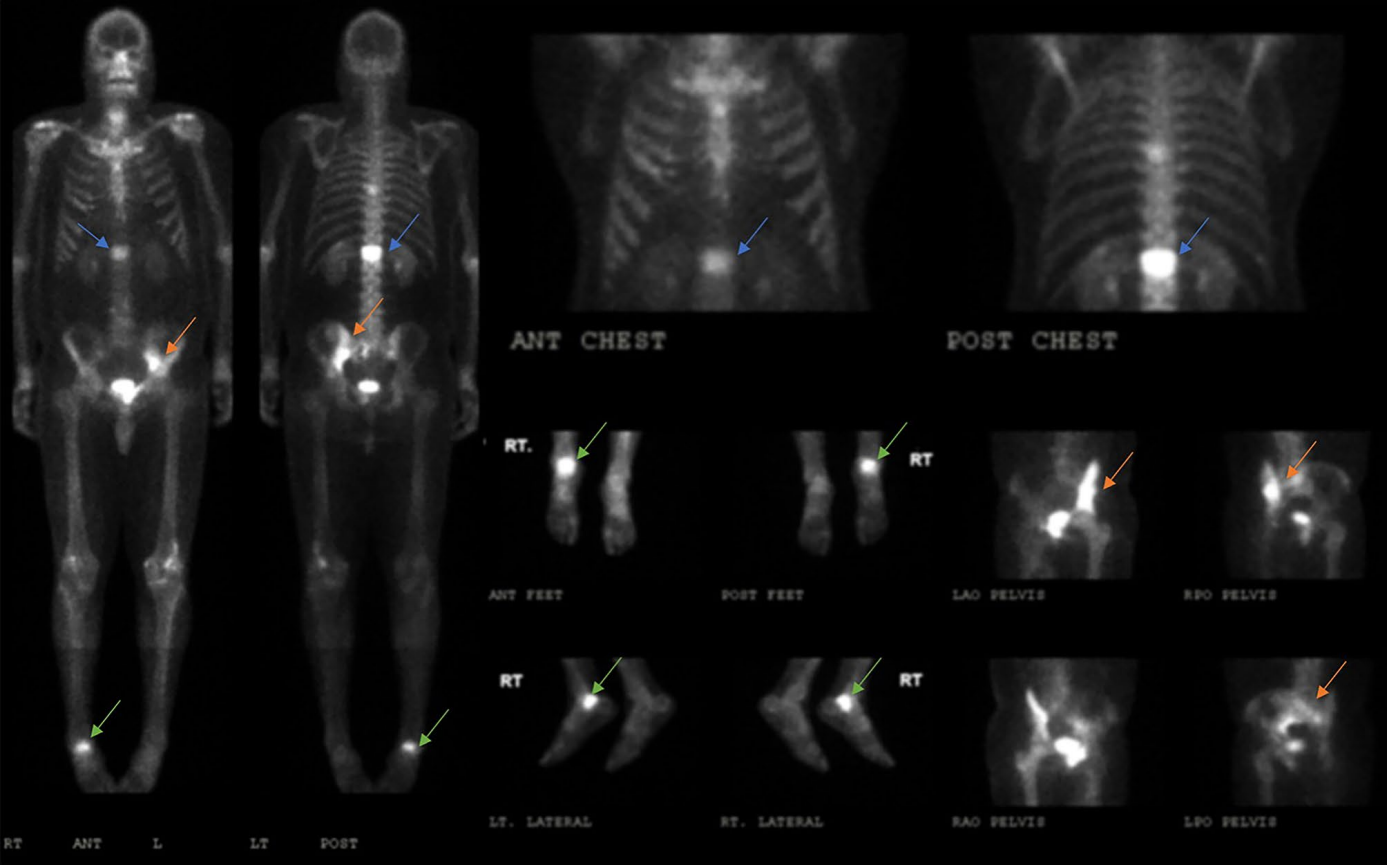
Whole body 99mTc-MDP bone scan and multiple spot views images of patient 2 (ID 2). A 43-years-old male with inclusion body myopathy, and previously diagnosed Paget’s disease revealed positive tracer uptake in the spine (blue arrow), left iliac bone (orange arrow) and right ankle (green arrow).

**Figure 3:**
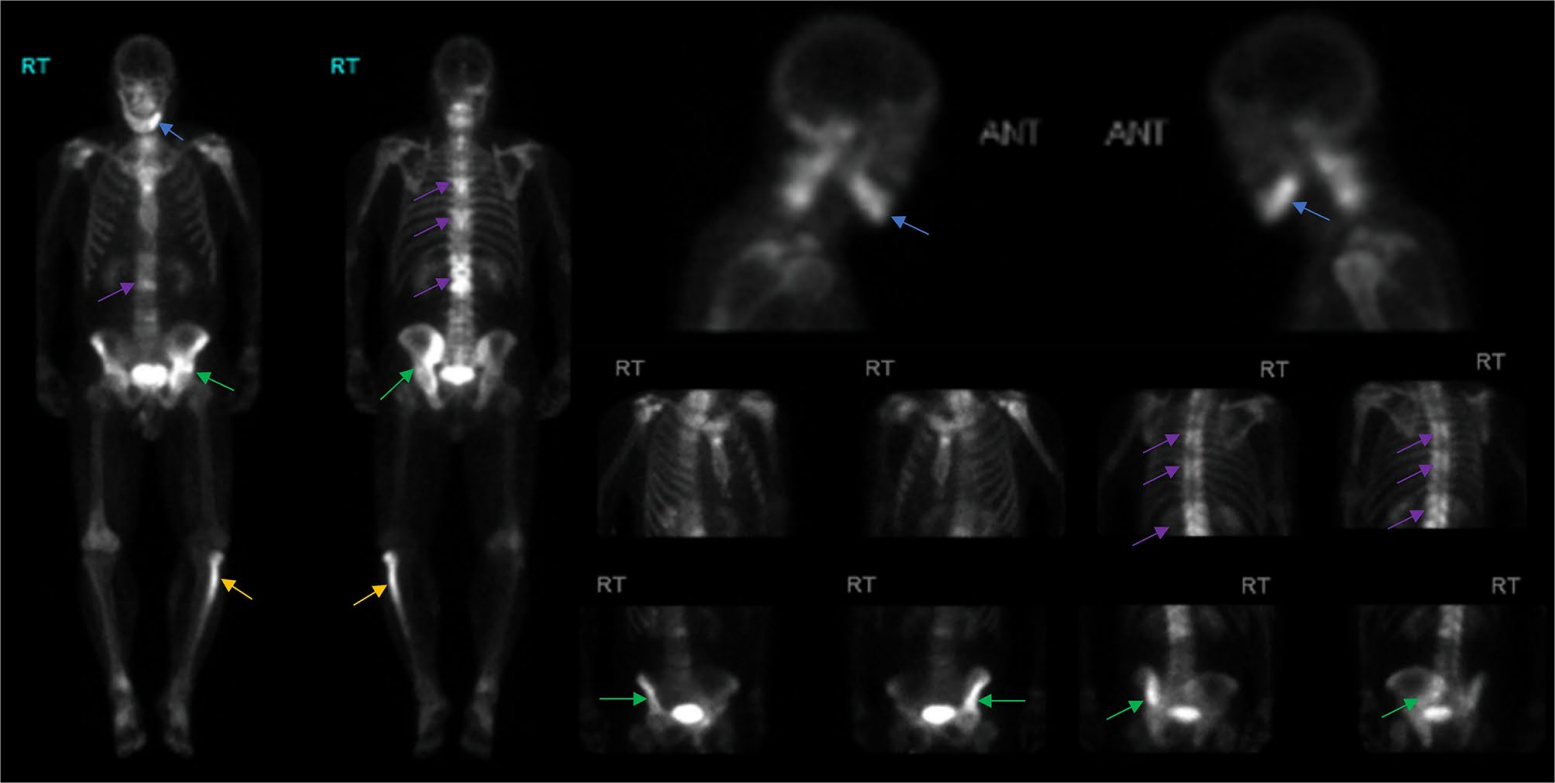
Whole body 99mTc-MDP bone scan images and multiple spot views of patient 4. A 49-years-old male with inclusion body myopathy, and Paget’s disease revealed positive bone scan findings of the left mandible (blue arrow), left hemipelvis (green arrow), multiple levels of the thoracic and lumbar spine (purple arrow) and proximal left fibula (yellow arrow).

Six (50%) MSP1 patients had spine and rib involvement (Figs. 1, 2 and 3). Three patients had thoracic spine involvement while two had lumbar spine involvement with abnormal focal uptakes. Cervical spine vertebral uptake was seen in one patient. All six patients had one or multiple abnormal focal rib uptake. One presymptomatic patient (ID 12) had increased uptake at the anterior aspect of the right seventh rib attributed to DJD and not considered PDB.

### Pelvis

**Figure 4:**
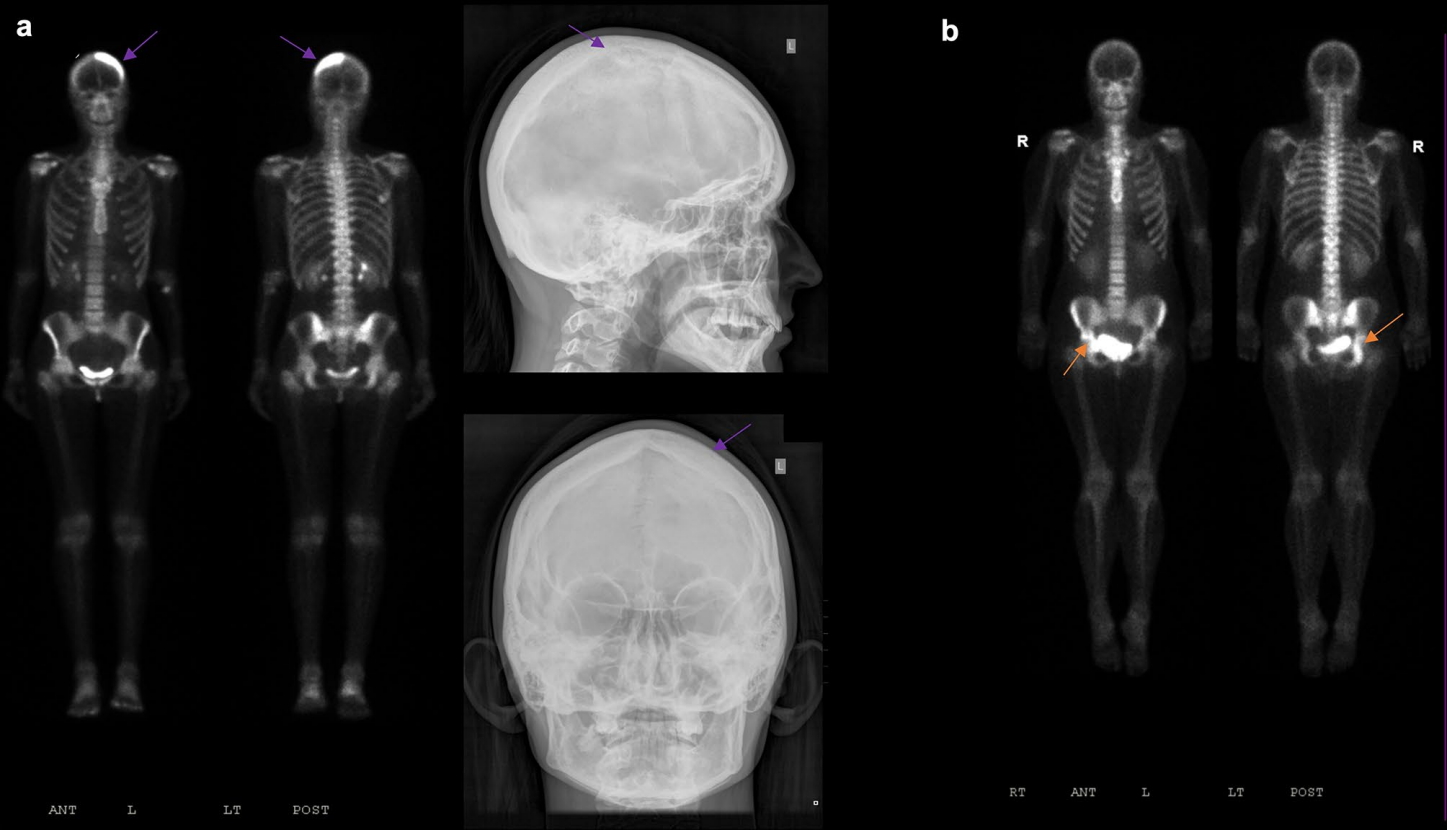
Whole body 99mTc-MDP bone scan and skull radiographs in two newly diagnosed PDB in myopathy only patients. (**a**) A 28-years-old female (ID 6) with increased uptake in the skull on the bone scan corresponding to a mixed lytic and sclerotic bone lesion in the left parietal calvarium on the AP and lateral views of the plain radiographs (purple arrows). (**b**) A 38-years-old female (ID 10) with increased uptake in the right ischium and acetabulum on whole-body bone scan (orange arrows).

Focal uptake in the pelvic region was seen in five (42%) MSP1 patients (Figs. 1, 2, 3 and 4). Acetabulum uptake was observed in three patients, and two had increased uptake in the ischium. Three patients had additional tracer uptake in hemipelvis areas including the sacrum, iliac crest and iliac brim, and superior pubic ramus.

### Skull

Calvarium uptake was observed in three (25%) MSP1 patients (Figs. 1 and 4a). Increased uptake was seen in the frontoparietal, parietal calvarium bones, and mandibles.

### Shoulder, clavicle, sternum, and manubrium

Five (42%) patients had increased tracer uptake in the shoulder and clavicle region. Increased activity was seen in the sternoclavicular joints in two patients. Three patients also had an increased radiotracer uptake in the manubriosternal joint. One presymptomatic patient had increased tracer uptake in the clavicle due to DJD.

### Lower extremity

Increased uptake in the lower extremity regions was observed in three (25%) MSP1 patients. One patient had increased uptake in the patella and ankle region (Fig. 2). One patient had increased uptake in the proximal femur, midfoot, and first toe. Another patient had increased uptake in the proximal left fibula (Fig. 3).

### Degenerative joint disorder (DJD) changes

Degenerative bone changes were noted on bone scan findings in three of the six patients with myopathy and PDB, three with myopathy only and one pre-symptomatic carrier. In PDB patients, DJD was seen in the lumbar and thoracic spine, clavicle, and scapula. DJD affected areas such as clavicle, bilateral shoulders, sternoclavicular joints, and left patella were seen in one pre-symptomatic carrier.

### Laboratory results

ALP levels were elevated in only one of the four patients (234 IU/L) who had myopathy and PDB at the time of visit, and three had normal values (mean 99.6 IU/L, normal 44–147 U/L). The newly diagnosed PDB patients also did not exhibit elevated ALP levels (mean 80 IU/L). Two patients with myopathy and PDB had elevated CPK levels (mean > 250 IU/L, normal 20–200 IU/L), all the other individuals had normal levels.

### Treatment

Half of our patients (6/12) were not taking any form of medications nor supplements. Among those taking medications 4/6 (66.7%) were receiving daily intake of multivitamins and only one (16.7%) was receiving bisphosphonate (ID 4, zoledronic acid). Other medications reported by patients included analgesics, anesthetics, anticholinergics, selective serotonin reuptake inhibitor, diuretics, and anti-lipidemics.

## Discussion

Typical manifestation of pathogenic variants of the *VCP* gene (MSP1) includes progressive axial and proximal muscle weakness, PDB starting in their thirties respectively, and FTD starting in their fifties^1^. Compared to conventional PDB with an approximate mean onset age of 50 years, MSP1 patients have an earlier mean age of onset of PDB in their late thirties as was noted in our cohort^15^. This is the first effort to formally analyze bone scan findings from MSP1 patients to ascertain and characterize PDB disease. Farpour et al. previously only assessed plain radiographs from MSP1 patients^22^.

Bone pain is typically the most common symptom (52–74%) followed by bone deformity (18–22%), deafness (8–9%) and fractures (6–9%) in PDB found in the general population^25^, however, we only found that one individual had associated pain in our cohort. Serum ALP level was previously considered the first line biochemical screening in high risk PDB population due to its widespread availability and affordability. However, serum ALP screening may not have high enough clinical sensitivity and specificity in the diagnosis of early or small Paget disease lesions^21^. As noted in our cohort of subjects, only one individual with active PDB were identified with an elevated ALP level.

Interestingly two myopathy patients were diagnosed with PDB as a result of the testing at the time of visit despite having normal ALP levels and no bone pain. PDB was observed in the left parietal calvarium in ID 6 while PDB was observed in the right ischium and acetabulum in ID 10 (Fig. 4). Subsequent plain radiograph imaging was performed after increased bone scan focal uptakes were observed. The increased radiotracer uptake of the affected bones found in our study such as the spine, pelvis, and skull are of typical distribution as seen in conventional PDB. Among the six patients diagnosed with PDB, three patients showed increased activity in the skull, none of which showed the ring-like pattern on the margin of the lesions typically seen in conventional PDB. Three MSP1 patients also had increased uptake of the lower extremities as was noted in other types of MSPs^26,27^. On radiographs, we observed both lytic and blastic or sclerotic lesions on the skull radiographs for patient 1 (ID 1), and more sclerotic appearance of the skull radiographs on patient 6 (ID 6).

Although these may be a consequence of aging, osteoarthritis is seen in approximately 73% of those suffering from general PDB, and commonly manifests in joints adjacent to the Pagetic bone^16^. In our study degenerative bone changes were noted on bone scan in three patients with PDB, three myopathy alone and one pre-symptomatic carrier. The degenerative changes were seen in the bone or a nearby bone in three patients in which PDB was seen. PDB may cause subchondral bone expansion leading to the narrowing of joint spaces and increased joint pressure promoting cartilage necrosis^17^. Our cohort also showed increased activity in the thoracic region: ribs, manubrium, and the sterno/acromio clavicular joints, which are commonly attributable to degenerative diseases^28,29^. The increased mechanical joint stress in osteoarthritis can induce osteoblastic response leading to asymmetrical Tc-99m uptake^18^. Studies have shown that trauma from sports, especially leading to non-union fractures can show persistent radiotracer uptake^30,31^. In addition, areas of repeated stress can show increased uptake such as the patellar tendon insertion to the tibial tuberosity^32^. Two of our patients (ID 7–8) reported trauma history however increased uptake in the relevant affected regions were excluded in the analysis.

There is currently no definitive treatment for the neuromuscular and neurological manifestations of MSP1. However, treatment with bisphosphonates such as alendronate and zoledronic acid has been noted to provide relief from bone pain in the majority of patients with PDB^14,33,34^. In our cohort, only one patient (ID 4) reported to be taking bisphosphonates who had five active PDB regions and an ALP level of 234 IU/L. Bone scans in addition to ALP levels can be effective in monitoring bone osteoclast reactivation in patients following treatment^34^. Future studies including bone-specific alkaline phosphatase (BALP), procollagen type I intact N-terminal propeptide (P1NP), and collagen type 1 C-telopeptide (CTX) may provide data on their usefulness in diagnosing and monitoring of PDB in this at-risk population^13^. Treatment comprises of bisphosphonates including zoledronic acid, pamidronate, risedronate and alendronic acid, and can help in mitigating symptoms of bone pain. Treatment response and recurrence can be monitored with raised ALP or increased uptake on bone scan. Vitamin D deficiency should be corrected prior to administration of bisphosphonates, to mitigate against hypocalcemia^14^. The ZiPP trial in presymptomatic patients with *SQSTM1* pathogenic variants will determine if early treatment with zoledronic acid can prevent PDB^35^. A similar treatment trial in *VCP* MSP1 patients has the potential to prevent the occurrence of PDB in this at-risk population.

Our study has some limitations. Due to the rarity of disease and burden of travel to our center, the cohort size is relatively small. In addition, MSP1 patients have significant variability and expressivity. Nevertheless, *VCP* MSP1 is a rare disease, and this series represents a significant addition to the literature. Recent studies have reported intergenerational change of PDB phenotype in general population and those with *SQSTM1* mutations^20,36^. In our study, we did not characterize these changes as our cohort is small although we anticipate a subsequent study characterizing the severity of PDB phenotype in MSP1 patients. As a technique, nuclear imaging can be time-consuming, but is useful in screening asymptomatic bone sites due to its high sensitivity.

Positive findings on bone scans, in conjunction with the clinical history and assessment and correlation with other imaging modalities such as skeletal radiography is considered very suspicious of the diagnosis of PDB in this high-risk population. Our study shows that bone scan imaging can be used in conjunction with clinical history, laboratory testing and plain radiographs to ascertain affected Pagetic bones in asymptomatic patients with MSP1 due to its high detection sensitivity. Early surveillance, treatment, and monitoring are paramount to prevent the serious complications of PDB in MSP1 patients.

## Data Availability

All data presented in this study are available from the corresponding author on request.
